# Protective effect of new histone deacetylase 6 inhibitors in a cisplatin-induced peripheral neurotoxicity murine model

**DOI:** 10.1097/PR9.0000000000001395

**Published:** 2026-01-30

**Authors:** Guido Cavaletti, Annalisa Canta, Alessia Chiorazzi, Elisa Ballarini, Virginia Rodriguez, Arianna Scuteri, Gabriella Nicolini, Alessio Malacrida, Paola Cordella, Andrea Stevenazzi, Barbara Vergani, Christian Steinkühler, Simonetta Andrea Licandro

**Affiliations:** a Experimental Neurology Unit, School of Medicine and Surgery, University of Milano-Bicocca, Monza, Italy; b Fondazione IRCCS San Gerardo dei Tintori di Monza, Monza, Italy; c Preclinical R&D Department, Italfarmaco S.p.A., Cinisello Balsamo, Italy

**Keywords:** Chemotherapy-induced peripheral neuropathy, Cisplatin, HDAC6 inhibitors, Preventive effect, Curative effect

## Abstract

**Introduction:**

Histone deacetylase 6 (HDAC6) inhibitors have shown effectiveness in preclinical models of chemotherapy-induced peripheral neuropathy (CIPN), a severe side effect of various antineoplastic drugs, with no available preventive or therapeutic treatments. This study presents in vivo results for ITF6464 and ITF6475, 2 HDAC6 inhibitors featuring a difluoromethyloxadiazole (DFMO), zinc-binding group ensuring selectivity for HDAC6.

**Objectives:**

This study investigated the potential effect of 2 new selective DFMO HDAC6 inhibitors in preventive and curative settings in a well-established CIPN model.

**Methods:**

The effectiveness of treatments was evaluated by dynamic test and histological analysis on dorsal root ganglia (DRG) and skin biopsy. The acetylation of tubulin was also investigated in sciatic nerve by western blot.

**Results:**

ITF6464 and ITF6475 administered at 12.5 mg/kg dose prevented cisplatin-induced mechanical allodynia (***P < 0.001 and **P < 0.01, respectively). Histologically, both ITF6464 and ITF6475 at all doses prevented damage in DRGs. ITF6464 at 6 mg/kg and ITF6475 at 1 mg/kg protected intraepidermal nerve fibers (IENF) from cisplatin-induced damage (*P < 0.05). After 2 cisplatin cycles, ITF6475 12.5 mg/kg was effective in reverting mechanical allodynia (***P < 0.001). ITF6475 reverted cisplatin-induced damage in DRGs and, at the highest dose, also reverted IENF damage (^○○^*P* < 0.01). Cisplatin induced a reduction in acetylated tubulin in the sciatic nerve of mice, and ITF6464 and ITF6475 at 12.5 mg/kg dose were able to significantly revert this condition.

**Conclusions:**

These studies highlight the potential of the new selective HDAC6 inhibitors ITF6464 and ITF6475 as promising treatments for CIPN.

## Abstract 2

Supplemental Digital Content is Available in the Text.

A new class of highly selective histone deacetylase 6 inhibitors could address an important unmet clinical need in both preventing and treating chemotherapy-induced peripheral neuropathy.

## 1. Introduction

Histone deacetylase 6 (HDAC6) belongs to the zinc-dependent HDAC family. Unlike class I HDACs, which are predominantly located in the nucleus and deacetylate both histone and nonhistone proteins, HDAC6 is mainly localized in the cytoplasm and plays only a minor role in gene regulation. Consequentially, its major biological targets are nonhistone proteins, such as α-tubulin, Foxp3, Hsp90, β-catenin, and others. Histone deacetylase 6 contributes in regulating cytoskeletal dynamics and shape maintenance,^4,7,16^ and mitochondria migration and trafficking.^4–6^ It was also proposed to be involved in several pathological conditions. Remarkably, HDAC6 knock-out mice are viable and apparently show no gross impairments,^23^ suggesting that HDAC6 inhibition should be well-tolerated.

Histone deacetylase 6 inhibitors (HDAC6is) are characterized by the presence of a zinc-binding group (ZBG), a functional group that chelates or strongly interacts with the zinc ion in the enzyme active site. The commonly used ZBG is hydroxamic acid, but it is associated with potential genotoxicity and suboptimal pharmacokinetic profile.

Recently, the discovery of the difluoromethyloxadiazole (DFMO) moiety as a new ZBG, contributed to the development of new generations of highly selective HDAC6is.^10,12^ Difluoromethyloxadiazole derivatives are mechanism-based inhibitors, with a nearly absolute selectivity for HDAC6 when compared with the other HDACs.^3,8^ These compounds are safe and nongenotoxic, so they are amenable to be developed also for non-oncology indications.

Chemotherapy-induced peripheral neuropathy (CIPN) is a severe and potentially dose-limiting complication of several widely used antineoplastic drugs. Cisplatin, a cornerstone of the treatment of different malignancies including lung, testis, ovarian cancers, and sarcomas, is among the most severely neurotoxic anticancer drugs. Recovery from cisplatin-induced peripheral neurotoxicity (PN) is often incomplete and it persists in 55% of patients up to 15 years after the end of treatment.^1^

No preventive or therapeutic treatments are available to limit the severity of cisplatin-related PN and this lack represents an important unmet clinical need.

Histone deacetylase 6 inhibitors were previously reported to be effective in a preclinical CIPN model. Krukowski et al.^9^ described preventive and curative efficacy of ACY-1083, a hydroxamate HDAC6 inhibitor, on cisplatin-induced PN, by enhancing mitochondrial transport in dorsal root ganglia (DRG). This model, established by Ta et al.,^19^ is representative of structural and functional changes mainly in the peripheral sensory neurons and DRG. Cisplatin cumulative dose (23 mg/kg) causes a transient weight loss in mice but regarding renal failure and survival it is safe; furthermore, it is close to the human therapeutic doses relative to body weight.^13,14^ However, molecules such as ACY-1083 suffer from suboptimal drug-like properties and selectivity. Histone deacetylase 6 inhibitors could also promote IL-10 production and signaling in DRG by inhibiting HDAC6 in macrophages^22^ and could influence tonic enkephalin-opioid receptors signaling in DRG to inhibit mechanical allodynia.^21^

ITF6464 and ITF6475 belong to a new DFMO-based class of HDAC6is^13^ with an almost absolute selectivity for HDAC6, and they are very potent on the isolated enzyme, showing a single digit nanomolar IC_50_, and potently induce a more than 20-fold increase in tubulin acetylation in cells. The 2 compounds are metabolically stable and show a favorable pharmacokinetic profile, with high AUCs and long half time. In addition, they gave very good results in *in vitro* CIPN models, a picture that prompted us to test ITF6464 and ITF6475 in vivo in preventive and curative settings.

## 2. Material and methods

### 2.1. Animal experiments

#### 2.1.1. Study approval

Procedures involving animals and their care were performed in conformity with institutional guidelines in compliance with national and international laws and policies (Italian Governing Law: D.lgs 26/2014 “Attuazione della direttiva 2010/63/UE sulla protezione degli animali utilizzati a fini scientifici”). The research project has been authorized by the Italfarmaco S.p.A. Animal Welfare Board and by the Italian Ministry of Health (Approval Number 303/2021-PR).

#### 2.1.2. Animals

C57BL/6J (Stock n°: 632) 7–8-week-old male mice^5,20^ were purchased from Charles River (Lyon, France). Mice were kept under pathogen-free conditions with a 12-h light/12-h dark cycle at a temperature of 22° ± 2° and 55% ± 10% humidity. All mice received water and chow (VRF1 diet, Charles River) ad libitum. Each cage was enriched with a mouse house and nesting material. Mice were regularly checked by a certified veterinarian who was responsible for health monitoring, animal welfare supervision, experimental protocols, and procedure revision. After 5 days of acclimatization in the animal facility, mice were randomized based on their body weight (BW) and dynamic plantar aesthesiometer test (dynamic test) values recorded before starting treatments (baseline time point) into 6 (Study 1 and 2) or 5 (Study 3) treatment groups (12 mice/group; 4 mice/cage) as summarized in Table 1.

**Table 1:** Experimental groups.

Study 1	Study 2	Study 3
Vehicle	Vehicle	Vehicle
Cisplatin 2.3 mg/kg	Cisplatin 2.3 mg/kg	Cisplatin 2.3 mg/kg
Cisplatin + ACY-1083 10 mg/kg	Cisplatin + ITF6464 12.5 mg/kg	Cisplatin + ACY-1083 10 mg/kg
Cisplatin + ITF6464 1 mg/kg	Cisplatin + ITF6475 1 mg/kg	Cisplatin + ITF6475 6 mg/kg
Cisplatin + ITF6464 6 mg/kg	Cisplatin + ITF6475 6 mg/kg	Cisplatin + ITF6475 12.5 mg/kg
Cisplatin + ITF6464 12.5 mg/kg	Cisplatin + ITF6475 12.5 mg/kg	

### 2.2. Drug treatment and experimental design

Pharmaceutical grade cisplatin (Teva Italia, Italy, 1 mg/mL) was diluted in sterile saline each injection day and administered by intraperitoneal (i.p.) injection at the dose of 2.3 mg/kg (5 days on/5 days off/5 days on, total cumulative dose = 23 mg/kg); ACY-1083 (MedChemTronica, Sollentuna, Sweden, Cat. N. HY-111791) was dissolved in 20% 2-hydroxypropyl-B-cyclodextrin (Roquette, Vecquemont, France) + 0.5% hydroxypropyl-methylcellulose (Sigma-Aldrich, Milan, Italy) in water each injection day and administered i.p. at the dose of 10 mg/kg (daily for 15 days); ITF6464 and ITF6475 were suspended in 0.5% methylcellulose (Sigma-Aldrich) every 5 days and were administered per os (p.o.) at the doses of 1, 6, and 12.5 mg/kg (daily for 15 days). Vehicle-treated mice received 0.5% methylcellulose p.o. (daily for 15 days) and 2-hydroxypropyl-B-cyclodextrin + 0.5% hydroxypropyl-methylcellulose i.p. (daily for 15 days). The administration volume for all drugs was 10 mL/kg. ACY-1083 was the reference compound in studies 1 and 3, while ITF6464 become the reference compound in study 2 after demonstrating its equivalence in preventive efficacy in study 1.

In studies 1 and 2, ACY-1083, ITF6464, and ITF6475 were administered together to cisplatin to prevent the chemo-induced peripheral damage, while in study 3, cisplatin was administered alone to induce peripheral nerve damage and after the second cycle of chemo, ACY-1083 and ITF6475 were administered to test the curative effect of HDAC6 inhibitors in this cisplatin-induced CIPN model (Table 2).

**Table 2:** Experimental design.

Experimental design									
Study 1 preventive setting	Cisplatin		XXXXX	XXXXX					
	ACY-1083/ITF6464		XXXXXXXXXXXXXXX						
	Dynamic test	X			X				
	Sacrifice				X				
Study 2 preventive setting	Cisplatin		XXXXX	XXXXX					
	ITF6464/ITF6475		XXXXXXXXXXXXXXX						
	Dynamic test	X			X				
	Sacrifice				X				
Study 3 curative setting	Cisplatin		XXXXX	XXXXX					
	ACY-1083/ITF6475				XXXXXXXXXXXXXXX				
	Dynamic test	X			X			X	
	Sacrifice								X

### 2.3. Neurotoxicity assessment

#### 2.3.1. Functional test

Dynamic test (Ugo Basile, Gemonio, Italy) was performed to verify the onset of chemotherapy-induced damage and allow the second randomization of mice (Table 2). This noninvasive functional test was performed at baseline and at the end of the experiment in study 1 and study 2 (preventive setting), while in study 3 (curative setting), the mechanical nociceptive threshold was also evaluated after the second cycle of cisplatin treatment. The dynamic test was performed by the same experimenter who was blinded to the treatments, as previously published.^2^ The results represented the maximal pressure (expressed in grams) tolerated by the animals. An upper limit cutoff of 20 seconds was set, after which the mechanical stimulus was automatically stopped to avoid stressing the animals.

### 2.4. Histological analysis

#### 2.4.1. Tissue samples collection

At the end of the treatment, mice were anesthetized with isoflurane (induction 3%–5%) and killed. L4-L5 DRG for morphometric analysis and skin biopsy for morphological and morphometric examination were collected from 4 mice/group.

#### 2.4.2. Pathological examination and dorsal root ganglia morphometric analysis

Pathological examination was performed on DRG after fixation in 4% paraformaldehyde/2% glutaraldehyde, followed by OsO_4_ postfixation and epoxy resin embedding. Semi-thin sections of 1.5 μm thick were prepared from at least 3 animals. For the morphometric analysis, DRG sections were stained with toluidine blue and examined with a Nexcope Ne920 AUTO (TEsseLab, Milan, Italy) light microscope at a magnification of ×20 and then analyzed with a computer-assisted image analyzer (Image J software, US National Institutes of Health). Serial sections spaced 25 μm were collected, and the somatic, nuclear, and nucleolar size of DRG were measured on at least 200 DRG/mouse in randomly selected sections spaced more than 50 μm. Only DRG with a nucleolar area larger than 2 µm was evaluated. Histopathological analyses were performed by a pathologist who was blinded to the treatments.

#### 2.4.3. Skin biopsy

To evaluate the intraepidermal nerve fiber (IENF) density, glabrous skin punches from the plantar hind paws were fixed in paraformaldehyde 2%, cryoprotected and serially cut in 20 μm-thick sections. Sections were immunostained with rabbit polyclonal antiprotein gene product 9.5 (PGP 9.5; ProteinTech, Manchester, United Kingdom) using a free-floating protocol. The total number of PGP 9.5-positive IENF crossing the dermal–epidermal junction was counted under a light microscope at ×40 magnification (Nexcope Ne920 AUTO, TEsseLab). Intraepidermal nerve fiber density was expressed as number IENF/length of epidermis (mm). Immunohistochemical analyses were performed by a pathologist who was blinded to the treatments.

### 2.5. Western blot assays

#### 2.5.1. Western blot analysis: tubulin acetylation and histone H3 acetylation

Frozen sciatic nerves from 4 mice/group were homogenized in Complete Lysis-M (Roche, Basilea, Swiss) containing 1X Protease inhibitor (Roche), 1X Phos-Stop (Roche) with a TissueLyser II instrument (Qiagen, Milan, Italy). The resulting homogenate were sonicated and then centrifuged at 18000*g* at 4°C for 10 minutes. Proteins were quantified by Bradford protein assay and equal amount of extracts were loaded on SDS-PAGE gel (4%–20% TGX stain-free gels, BioRad, Hercules, CA). After electrophoresis, proteins were transferred to polyvinylidene difluoride filters for immunoblotting analysis following manufacturer instructions. Filters were incubated overnight with primary antibodies (anti-Acetylated Tubulin, 1:1000, Sigma-Aldrich, Saint Louis, MO T6793; anti-α-Tubulin, 1:1000, Cell Signaling, Leiden, Netherlands 2144; anti-Acetylated H3K27, 1:1000, Cell Signaling 8173; anti H3, 1:1000, Cell Signaling 14269; antivinculin; 1:1000, Santa Cruz, CA 73614). Afterward, filters were incubated for 1 hour with fluorescent secondary antibodies (goat antimouse IgG, Cy3 conjugated, 1:2500 and goat anti-rabbit IgG, Cy5 conjugated, 1:2500, Amersham ECL Plex). Images of filters were obtained with an Amersham Imager 600, and intensity of bands were quantified using ImageJ software.

### 2.6. Statistical analysis

Statistical analysis was performed using GraphPad Prism version 10 software (GraphPad Software, San Diego, CA). Body weight statistical analysis was performed by 2-way ANOVA with Bonferroni multicomparison test; differences in dynamic test and IENF density were statistically analyzed by 1-way ANOVA, Kruskal–Wallis, and Dunn post-test; differences in DRG morphometrical analysis and in immunoblotting analysis were statistically analyzed by parametric 1-way ANOVA test and Tukey post-test. *P* values ≤0.05 were considered as statistically significant and are indicated in each figure legend.

## 3. Results

### 3.1. Tolerability of treatments

**Figure 1.**
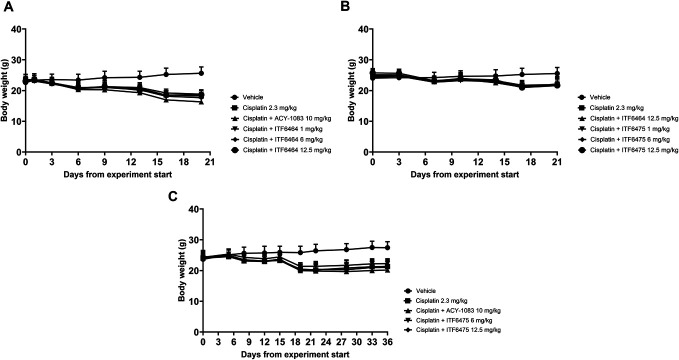
Body weight. (A) Study 1 (ITF6464 preventive effect). Statistical analysis: 2-way ANOVA with Bonferroni multiple comparison test vs vehicle; ***P < 0.001 (cisplatin 2.3 mg/kg, cisplatin + ITF6464 1 mg/kg, cisplatin + ITF6464 6 mg/kg and cisplatin + ITF6464 12.5 mg/kg) and ****P < 0.0001 (cisplatin + ACY-1083 10 mg/kg) at day 6; ****P < 0.0001 for all groups starting from day 9 until the end of the study (day 20). Data are represented as mean ± SD, n = 12 except for cisplatin + ACY-1083 group n = 11 (from day 16). (B) Study 2 (ITF6475 preventive effect). Statistical analysis: 2-way ANOVA with Bonferroni multiple comparison test. *P < 0.05 (cisplatin + ITF6475 1 mg/kg vs vehicle) and **P < 0.01 (cisplatin + ITF6464 12.5 mg/kg vs vehicle) at day 14; ****P < 0.0001 vs vehicle for all groups starting from day 17 until the end of the study (day 21). Data are represented as mean ± SD, n = 12. (C) Study 3 (ITF6475 curative effect). Statistical analysis: 2-way ANOVA with Bonferroni multiple comparison test. *P < 0.05 (cisplatin + ACY-1083 10 mg/kg and cisplatin + ITF6464 12.5 mg/kg vs vehicle), **P < 0.01 (cisplatin 2.3 mg/kg vs vehicle) at day 8; **P < 0.01 (cisplatin 2.3 mg/kg, cisplatin + ACY-1083 10 mg/kg and cisplatin + ITF6464 12.5 mg/kg vs vehicle) at day 12; *P < 0.05 (cisplatin + ITF6464 12.5 mg/kg vs vehicle) and **P < 0.01 (cisplatin 2.3 mg/kg, cisplatin + ACY-1083 10 mg/kg vs vehicle) at day 15; ****P < 0.0001 vs vehicle for all groups starting from day 19 until the end of the study (day 36). Data are represented as mean ± SD, n = 12.

After the first and second cisplatin treatment cycles, a significant decrease in mean BW was observed in all cisplatin-treated mice compared with vehicle-treated animals starting from day 6, 14, and 8 in study 1, study 2, and study 3, respectively (Figs. 1A–C, respectively). Nevertheless, all mice maintained grooming habits and there was no evidence of severe general toxicity. All treated mice survived until the end of the studies, except for 1 mouse treated with cisplatin + ACY-1083 10 mg/kg that was killed at day 16 due to 20% BW loss lasting more than 72 hours (Study 1).

### 3.2. Study 1

#### 3.2.1. Functional tests

**Figure 2.**
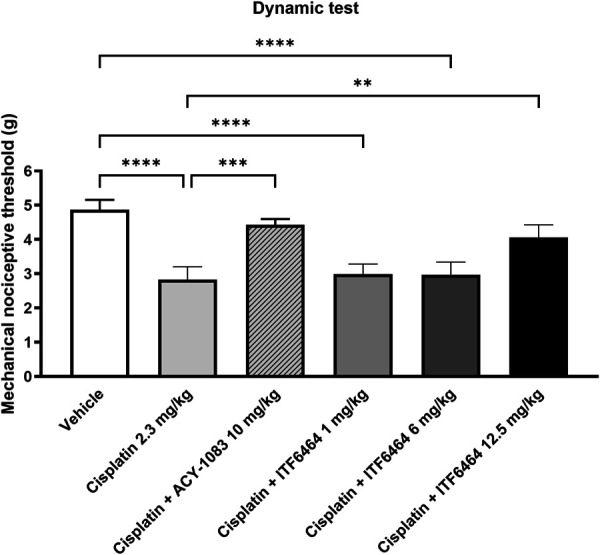
Dynamic test post-treatment (Study 1). Statistical analysis: 1-way ANOVA, Kruskal–Wallis, Dunn post-test; **P < 0.01, ***P < 0.001, ****P < 0.0001. Data are represented as mean ± SD, n = 12, except for cisplatin + ACY-1083 group n = 11.

At baseline, there were no statistically significant differences among experimental groups in mechanical allodynia as evaluated by the dynamic test (data not shown). After the second treatment cycle, cisplatin-induced mechanical allodynia was detectable in all treated mice (****P < 0.0001 vs vehicle-treated mice) and both ACY-1083 at 10 mg/kg and ITF6464 at 12.5 mg/kg prevented this occurrence (***P < 0.001 and **P < 0.01 vs cisplatin-treated mice, respectively) (Fig. 2).

#### 3.2.2. Histological analysis

##### 3.2.2.1. Dorsal root ganglia morphometric analysis

**Figure 3.**
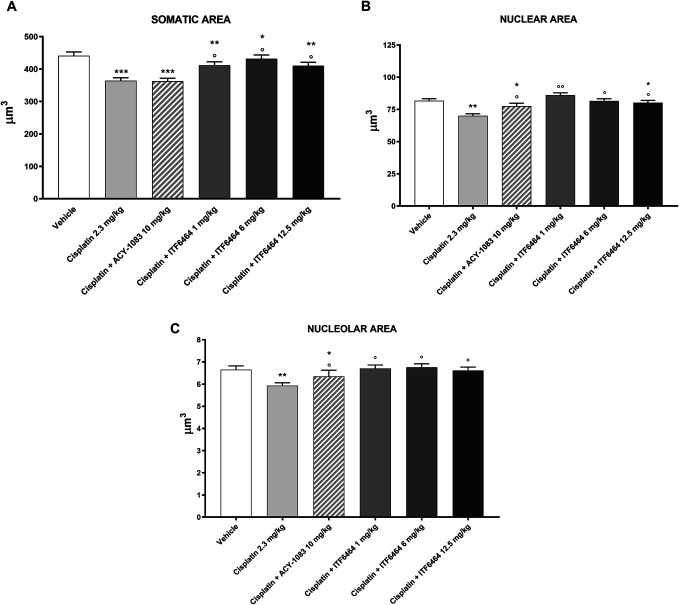
Dorsal root ganglia (DRG) morphometric analysis evaluated at the end of treatment (Study 1). Statistical analysis of somatic (A), nuclear (B), and nucleolar (C) areas of DRG was performed by nonparametric 1-way ANOVA test, Kruskal–Wallis, Dunn post-test; *P < 0.05, **P < 0.01, ***P < 0.001 vs vehicle, ^○^*P* < 0.05, ^○○^*P* < 0.01 vs cisplatin (n = 3, 4 DRG/mouse).

At the end of treatment, groups receiving cisplatin alone or in combination with ACY-1083 showed a statistically significant reduction in somatic, nuclear, and nucleolar areas in comparison with the vehicle group (Figs. 3A–C, respectively). ITF6464-treated groups showed a statistically significant reduction in somatic area vs vehicle-treated group (Fig. 3A). In addition, a reduction in nuclear area was observed in animals treated with ITF6464 at the dose of 12.5 mg/kg when compared with the vehicle-treated group (Fig. 3B). Nuclear and nucleolar compartments of DRG of mice treated with cisplatin + ITF6464 1 mg/kg and 6 mg/kg were preserved when compared with DRG of the cisplatin-treated group (Figs. 3B and C). ITF6464 at all doses was able to significantly counteract the reduction of somatic, nuclear, and nucleolar areas of DRG when compared with cisplatin-treated mice (Figs. 3A–C, respectively).

##### 3.2.2.2. Intraepidermal nerve fiber density analysis

**Figure 4.**
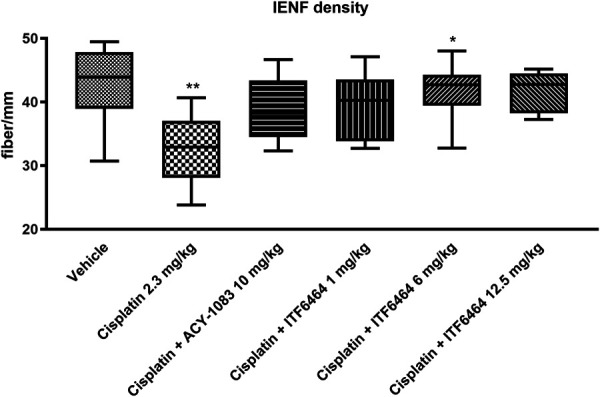
Intraepidermal nerve fiber (IENF) density evaluated at the end of treatment (Study 1). Data are depicted with box-and-whiskers plots and represent median (line of the box), 25th percentile (bottom line of the box), and 75th percentile (top line of the box) minimum and maximum values (end of the whiskers). Statistical analysis: nonparametric 1-way ANOVA test, Kruskal–Wallis, Dunn post-test; *P < 0.05 vs cisplatin, **P < 0.01 vs Vehicle.

At the end of treatment, a statistically significant reduction in IENF density was observed in mice treated with cisplatin alone compared with vehicle-treated mice. A statistically significant increase in IENF density was observed in cisplatin + ITF6464 6 mg/kg cotreated mice vs cisplatin-treated mice. In the other cotreatment groups, no statistically significant modulation was observed, neither vs vehicle nor vs cisplatin (Fig. 4).

### 3.3. Study 2

#### 3.3.1. Functional test

**Figure 5.**
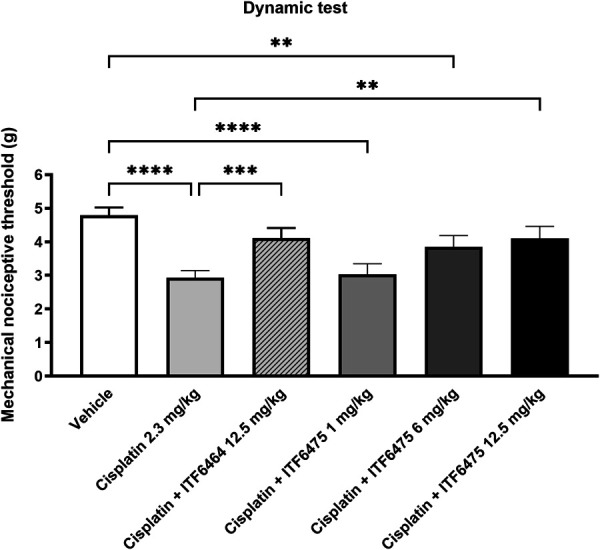
Dynamic test post-treatment (Study 2). Statistical analysis: 1-way ANOVA, Kruskal–Wallis, Dunn post-test; **P < 0.01, ***P < 0.001, ****P < 0.0001. Data are represented as mean ± SD, n = 12.

Based on the previous results, in this study, ITF6464 at 12.5 mg/kg dose was used as a reference compound. Cisplatin induced mechanical allodynia in all treated mice (****P < 0.0001 vs vehicle-treated mice), as observed in study 1. Remarkably, both ITF6464 and ITF6475 at 12.5 mg/kg were able to prevent this occurrence (***P < 0.001 and **P < 0.01 vs cisplatin-treated mice, respectively). In addition, we observed a dose-dependent protective effect of ITF6475 on peripheral damage (Fig. 5).

#### 3.3.2. Histological analysis

##### 3.3.2.1. Dorsal root ganglia morphometric analysis

**Figure 6.**
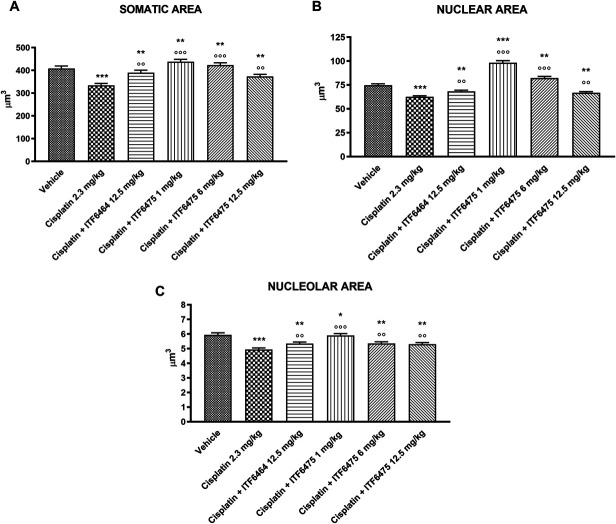
Dorsal root ganglia (DRG) morphometric analysis evaluated at the end of treatment (Study 2). Statistical analysis of somatic (A), nuclear (B), and nucleolar (C) areas of DRG was performed by nonparametric 1-way ANOVA test, Kruskal–Wallis, Dunn post-test; *P < 0.05, **P < 0.01, ***P < 0.001 vs vehicle; ^○○^*P* < 0.01, ^○○○^*P* < 0.001 vs cisplatin (n = 4).

At the end of treatment, a statistically significant reduction in somatic, nuclear, and nucleolar areas was observed in cisplatin vs vehicle-treated mice (Figs. 6A–C, respectively). ITF6475 at 1 and 6 mg/kg was able to significantly counteract cisplatin-induced damage in somatic and nuclear areas of DRG when compared with vehicle-treated mice (Figs. 6A and B). ITF6464 12.5 mg/kg and ITF6475 at all doses were able to significantly counteract the reduction of somatic, nuclear, and nucleolar areas of DRG when compared with the cisplatin-treated group (Figs. 6A–C, respectively).

##### 3.3.2.2. Intraepidermal nerve fiber density analysis

**Figure 7.**
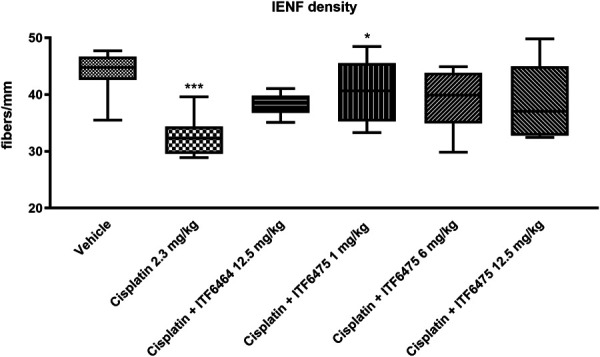
Intraepidermal nerve fiber (IENF) density evaluated at the end of treatment (Study 2). Statistical analysis: nonparametric 1-way ANOVA test, Kruskal–Wallis, Dunn post-test; *P < 0.05 vs cisplatin, ***P < 0.001 vs vehicle (n = 4).

A statistically significant reduction in IENF density vs vehicle was observed in animals treated with cisplatin, whereas all cotreatment groups were indistinguishable from vehicle. We also notice that only the cisplatin + ITF6475 1 mg/kg–treated group showed a statistically significant increase in IENF density when compared with cisplatin-treatment alone (Fig. 7).

#### 3.3.3 Western blot analysis

##### 3.3.3.1. Tubulin acetylation and histone H3 acetylation

**Figure 8.**
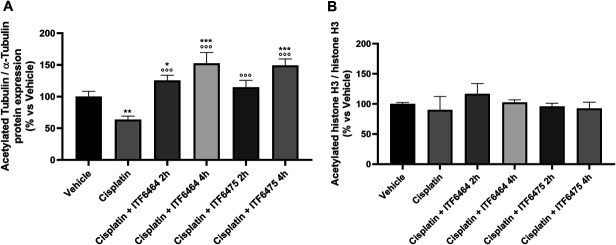
Tubulin (A) and Histone H3 immunoblotting (B) at the end of treatment (Study 2). Statistical analysis: parametric 1-way ANOVA test, Tukey post-test; *P < 0.05, **P < 0.01, ***P < 0.001 vs vehicle and ^○○○^*P* < 0.001 vs cisplatin (n = 4).

To ascertain whether HDAC6i treatment led to pharmacodynamic changes in nerve tissue, the acetylation state of tubulin and histone H3 (H3) in sciatic nerve was investigated by western blot. The results obtained showed that cisplatin induced a reduction in acetylated tubulin in the sciatic nerve, and both DFMOs at a dose of 12.5 mg/kg were able to significantly revert this tubulin acetylation decrease after 2 and 4 hours from treatment start (Fig. 8A and Fig. 1S, http://links.lww.com/PR9/A374). On the other hand, western blot analysis did not show any statistically significant modulation of H3 acetylation status by cisplatin and either HDAC6 inhibitor (Figs. 8B and Fig. 1S, http://links.lww.com/PR9/A374).

From the data obtained in both studies 1 and 2, we can conclude that 2 different DFMO-based HDAC6 inhibitors were able to effectively and dose-dependently prevent cisplatin-induced neuropathy in this CIPN murine model based on functional and pathologic evidence.

### 3.4. Study 3

#### 3.4.1. Functional test

In this experimental setting, mice received 2 treatment cycles of cisplatin. After the last administration, they were randomized into different experimental groups to receive ITF6475 (6 and 12.5 mg/kg doses) or ACY-1083, used as a reference.

**Figure 9.**
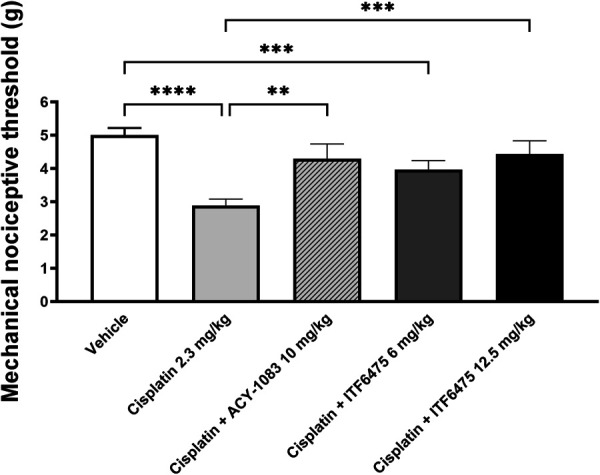
Dynamic test post-treatment (Study 3). Statistical analysis: 1-way ANOVA, Kruskal–Wallis, Dunn post-test; **P < 0.01, ***P < 0.001, ****P < 0.0001. Data are represented as mean ± SD, n = 12.

Mechanical allodynia was evident in all mice after the second cycle of cisplatin treatment (****P < 0.0001 vs vehicle-treated mice, data not shown). Both ACY-1083 10 mg/kg and ITF6475 at 12.5 mg/kg were able to revert cisplatin-induced damage (**P < 0.01 and ***P < 0.001 vs cisplatin-treated mice, respectively). In addition, in this experiment, ITF6475 was efficacious in a dose-dependent manner (Fig. 9).

#### 3.4.2. Histological analysis

##### 3.4.2.1. Dorsal root ganglia morphometric analysis

**Figure 10.**
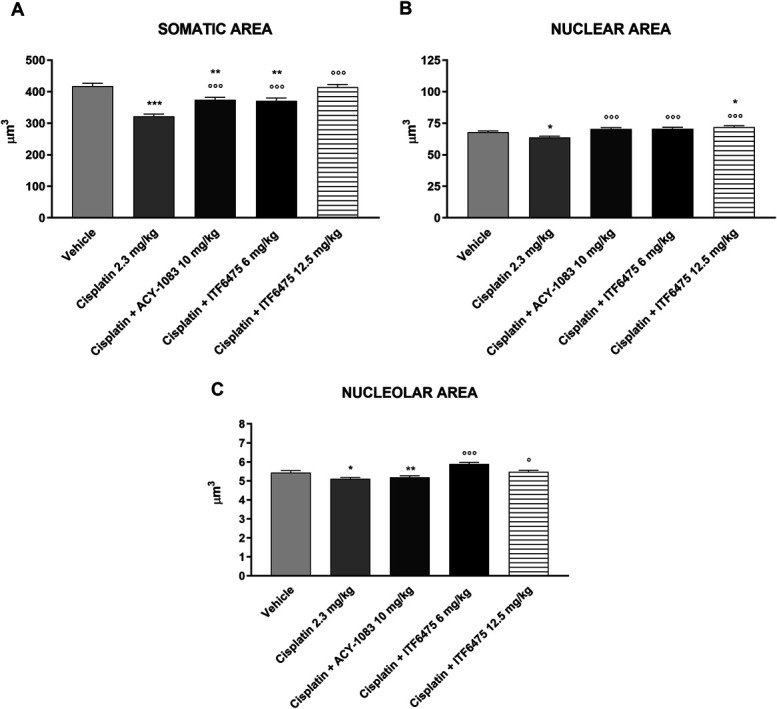
Dorsal root ganglia (DRG) morphometric analysis evaluated at the end of treatment (Study 3). Statistical analysis of somatic (A), nuclear (B), and nucleolar (C) areas of DRG was performed by nonparametric 1-way ANOVA test, Kruskal–Wallis, Dunn post-test; *P < 0.05, **P < 0.01, ***P < 0.001 vs vehicle; ^○^*P* < 0.05, ^○○○^*P* < 0.001 vs cisplatin (n = 4).

At the end of treatment, a statistically significant reduction in somatic, nuclear, and nucleolar areas of DRGs was observed in cisplatin vs vehicle-treated groups (Figs. 10A–C, respectively). A reduction in somatic area was also observed in cisplatin + ITF6475 6 mg/kg–treated mice when compared with vehicle-treated mice (Fig. 10A), while mice treated with ACY-1083 showed a statistically significant reduction in both somatic and nucleolar area of DRG when compared with vehicle-treated mice (Figs. 10A and C). ITF6475 at 12.5 mg/kg administered after the second cycle of cisplatin treatment significantly reverted cisplatin-induced damage of somatic, nuclear, and nucleolar areas of DRG if compared with cisplatin-treated group (Figs. 10A–C, respectively).

##### 3.4.2.2. Intraepidermal nerve fiber density analysis

**Figure 11.**
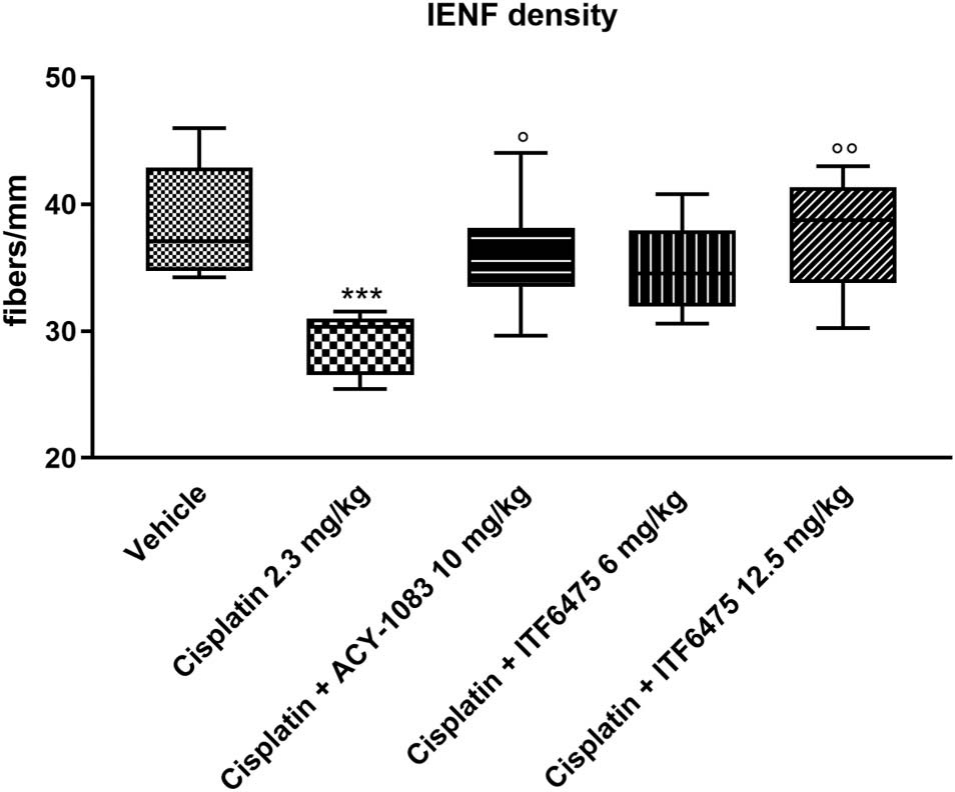
Intraepidermal nerve fiber density evaluated at the end of treatment (Study 3). Statistical analysis: nonparametric 1-way ANOVA test, Kruskal–Wallis, Dunn post-test; ***P < 0.001 vs vehicle and ^○^*P* < 0.05, ^○○^*P* < 0.01 vs cisplatin (n = 4).

Cisplatin treatment led to a statistically significant reduction in IENF density that was reverted on HDAC6 inhibitors treatment. Furthermore, cisplatin + ACY-1083 10 mg/kg and cisplatin + ITF6475 12.5 mg/kg cotreated groups also showed a statistically significant increase in IENF density vs cisplatin-treated group (Fig. 11).

#### 3.4.3. Western blot analysis

##### 3.4.3.1. Tubulin acetylation and histone H3 acetylation

The acetylation state of tubulin and histone H3 (H3) in sciatic nerve of mice was investigated by western blot.

**Figure 12.**
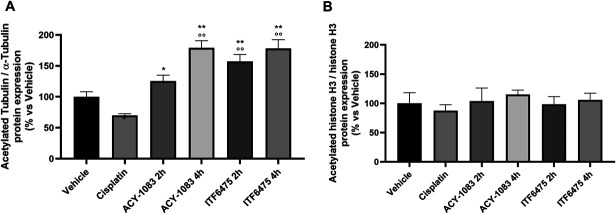
Tubulin (A) and Histone H3 immunoblotting (B) at the end of treatment (Study 3). Statistical analysis: parametric 1-way ANOVA test, Tukey post-test; *P < 0.05, **P < 0.01 vs vehicle and ^○○^*P* < 0.01 vs cisplatin (n = 4).

According to study 2, cisplatin induced a reduction of acetylated tubulin levels that was countered by both HDAC6 inhibitors. We noticed that ITF6475 12.5 mg/kg seemed to outperform ACY-1083 10 mg/kg after 2 hours post-treatment (Fig. 12A and Fig. 2S, http://links.lww.com/PR9/A374). In line with the selectivity of the compounds investigated, no statistically significant modulation of histone H3 acetylation status was observed (Fig. 12B and Fig. 2S, http://links.lww.com/PR9/A374).

We can conclude that ITF6475 DFMO-based HDAC6 inhibitor was also able to reverse cisplatin-induced neuropathy in this CIPN murine model.

## 4. Discussion

Chemotherapy-induced peripheral neuropathy–related symptoms such as sensory loss, paresthesia, dysesthesia, and numbness, often aggravated by neuropathic pain, may become dose-limiting toxicities for patients undergoing chemotherapy. Platin-induced peripheral neuropathy is a sensory neuronopathy determined by primary damage to DRG neurons eventually leading to axonal degeneration. Dorsal root ganglia are vascularized by fenestrated capillaries that render them more accessible to circulating compounds, including exogenous toxic substances such as platin-derived drugs. Moreover, DRG sensory neurons express transporters that enhance cell entry of platin-compounds and were shown to contain low levels of glutathione that participates in the inactivation of platin-drugs.^17^ These factors may therefore add to confer high susceptibility to platin-induced damage of DRGs.

Mechanistically, platin was proposed to cause morphological alterations in DRG nucleoli as a consequence of DNA damage induced by platin–adduct formation.^1^ Subsequently, mitochondria have emerged as an important factor in the development of CIPN. Mitochondria are deficient in classical DNA repair pathways making them more susceptible to the toxic action of platin drugs, and mitochondrial dysfunction might lead to impaired energy supply and oxidative stress. Moreover, impaired trafficking of mitochondria between the neuronal body and the periphery through the axonal transport has been suggested as a potentially relevant cisplatin-related neurotoxicity mechanism.^6,15^

Histone deacetylase 6 activity negatively affects axonal transport of mitochondria at least at 2 levels: (1) through deacetylation of alpha tubulin and (2) through deacetylation of the cargo protein MIRO1. Through its deacetylase activity, HDAC6 weakens the interaction between mitochondria and kinesin motor proteins, ultimately affecting mitochondria transport. Interestingly, an increase in HDAC6 activity was described to occur in CIPN, leading to a reduced axonal transport of mitochondria.^11^ These data suggest that decreasing HDAC6 activity is expected to be beneficial to limit the severity of CIPN.

Histone deacetylase 6 inhibition has been described to decrease cisplatin-induced PN in preclinical models.^9^ However, those studies were conducted using hydroxamic acid inhibitors, such as ACY-1083, with poor drug-like properties that show residual inhibitory activity on the other HDACs and may have additional off-target activities.^14^ To ascertain whether truly HDAC6-selective pharmacological inhibition is neuroprotective in a cisplatin-induced PN model, we tested ITF6464 and ITF6475 in 3 distinct preclinical studies. These compounds inhibit HDAC6 with an IC_50_ = 5.0 and 4.3 nM, respectively, and no detectable inhibitory activity on the other HDACs was observed. The 2 molecules induced higher levels of tubulin acetylation in 697 B-cell precursor leukemia line compared with ACY-1083 (EC50 = 183 nM, 119 nM, and 36 nM for ACY-1083, ITF6464, and ITF6475, respectively) and in SH-SY5Y neuroblastoma cells (data not shown). Despite their high protein binding in mouse plasma (98% for ITF6464 and 99% for ITF6475), active concentrations could be reached at relatively low doses, due to favorable PK profile: after an oral administration of 5 mg/kg, ITF6475 reached a Cmax of 1.8 µg/mL 6 hours after dose and an AUCtot of approximately 15 µg × h/mL.^2^ ITF6464 showed very similar PK properties. Their safety profiles are good as well, as they are Ames negative and did not show any sign of toxicity both in vitro and in vivo in the tested dose range.

In this study, we demonstrated that ITF6464 and ITF6475 prevent and reverse cisplatin-induced PN in a well-established CIPN murine model.^20^ The choice of the most suitable model can lead to a higher translational value of preclinical results for identifying promising treatments for CIPN. For this reason, in these studies, C57BL/6J male mice were used as per Ta et al's.^20^ published work. Furthermore, Gadgil et al.,^5^ performed a systematic review of the existing CIPN animal models and C57BL/6J male mice emerged as the most suitable model to study cisplatin-induced peripheral neuropathy. In the preventive setting, ITF6464 and ITF6475 were able to avoid the onset of mechanical allodynia, preserve the IENF density, and protect DRGs from cisplatin-induced toxicity. We also demonstrated that, in the curative setting, ITF6475 was able to reverse mechanical allodynia, restore the IENF density, and heal the DRG damage induced by cisplatin treatment. These results were comparable with those of ACY-1083, although ACY-1083 showed a more modest effect in both the behavioral test and in the histological analysis in both preventive and curative settings.

The selective inhibition of HDAC6 promoted α-tubulin acetylation in sciatic nerve with a consequent stabilization of microtubules. Through this mechanism, it is likely that the mitochondrial axonal transport is protected from the negative effect of cisplatin.^4,6,15^

Both CIPN prevention and reversal or amelioration of late-onset CIPN are clinically relevant. The treatment of established CIPN has the objective of improving the patient's quality of life, while the prevention setting has the potential to allow patients to get the full dose of chemotherapy and to complete all dosing cycles. However, preventive treatment must robustly demonstrate the lack of pharmacodynamic and/or pharmacokinetic interactions with the chemotherapeutic agent, that could potentially lead to interference with efficacy. It should be noted that HDAC inhibitors were originally developed as putative anticancer agents, leading to the approval by FDA of vorinostat for the treatment of cutaneous T-cell lymphoma; therefore, the issue of noninterference with concurrent anticancer treatment should not be particularly relevant.^18^ Another important aspect of the use of neuroprotective agents in combination with anticancer drugs is related to the possible increase of organ toxicities, making high target selectivity of the neuroprotective drug of utmost importance. Off-target inhibition of class I HDACs, as may occur with less selective hydroxamate-based HDAC6 inhibitors,^14^ may be highly problematic, as these enzymes are involved in processes such as cell cycle progression or DNA repair that may lead to interferences with both efficacy and tolerability of chemotherapy. We therefore believe that molecules such as our DFMOs that show almost absolute selectivity for HDAC6 over other HDAC subtypes are best suited for the development in a preventive CIPN setting.

In conclusion, these studies highlight the potential of the selective HDAC6 inhibitors ITF6464 and ITF6475 as promising treatments for CIPN. These compounds specifically target HDAC6, reducing the risk of side effects from off-target activity. They are effective in preventing and reducing nerve damage caused by cisplatin in animal models, showing clear improvements in nerve function and structure. In addition, the inhibitors are well-tolerated, with favorable pharmacokinetic properties, metabolic stability, and no signs of mutagenicity. These results suggest that ITF6464 and ITF6475 could address an important unmet need in preventing and treating CIPN. Further studies are needed to confirm their safety and effectiveness in humans and to explore their potential for other related conditions.

## Disclosures

S.A.L., P.C., C.S., B.V., and A.S. are Italfarmaco S.p.A employees. The other authors have no conflicts of interest to declare.

## Supplemental digital content

Supplemental digital content associated with this article can be found online at http://links.lww.com/PR9/A374.

## Supplementary Material

SUPPLEMENTARY MATERIAL
