# Supplementary material for: Protective effect of new histone deacetylase 6 inhibitors in a cisplatin-induced peripheral neurotoxicity murine model
Source: Pain Rep. 2026 Jan 30;11(2):e1395. doi: 10.1097/PR9.0000000000001395 (PMC12863917; doi:10.1097/PR9.0000000000001395)
Supplement: SUPPLEMENTARY MATERIAL [file painreports-11-e1395-s001.pdf]

Supplementary Materials

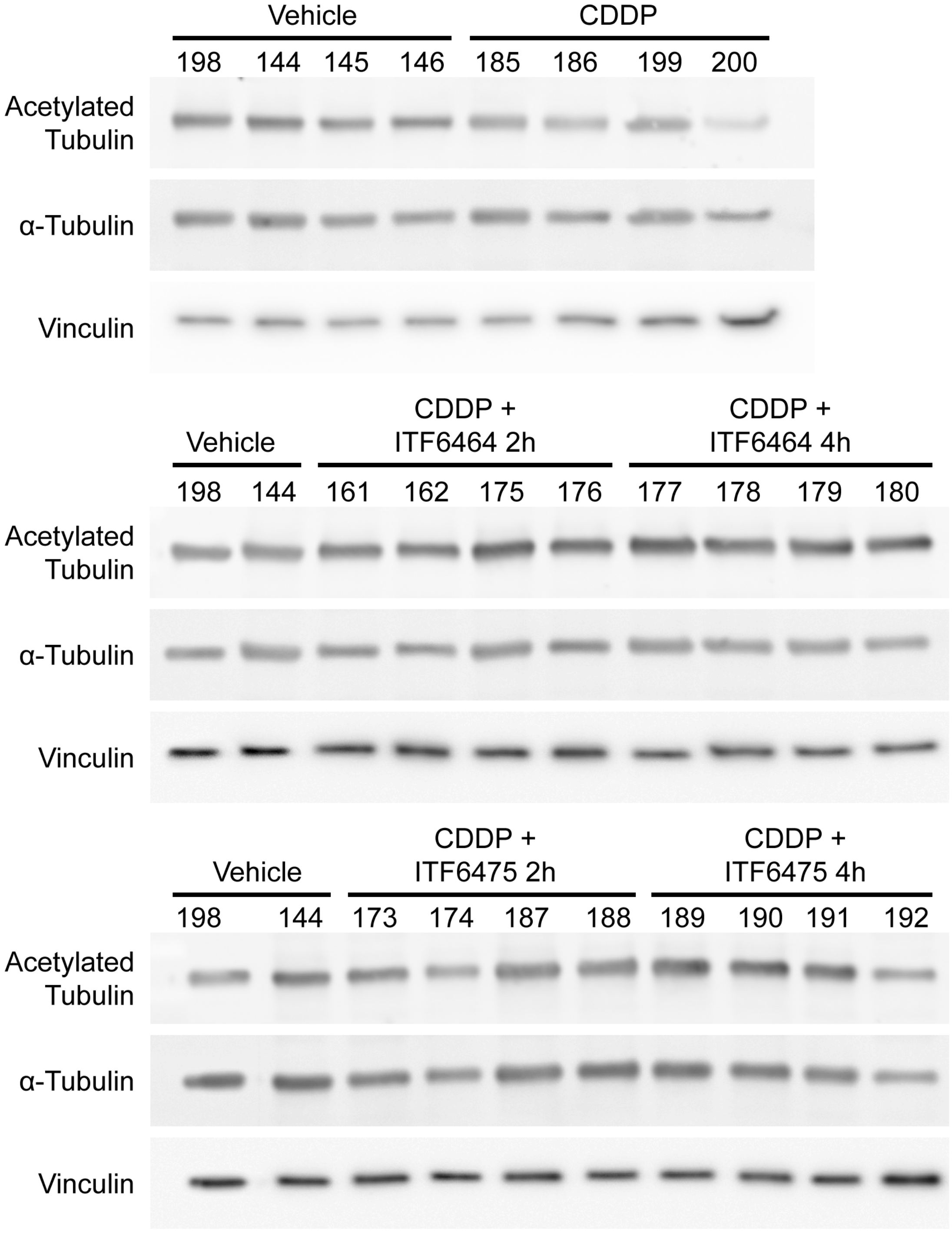

Figure 1S. Original western blot referred to Figure 8.

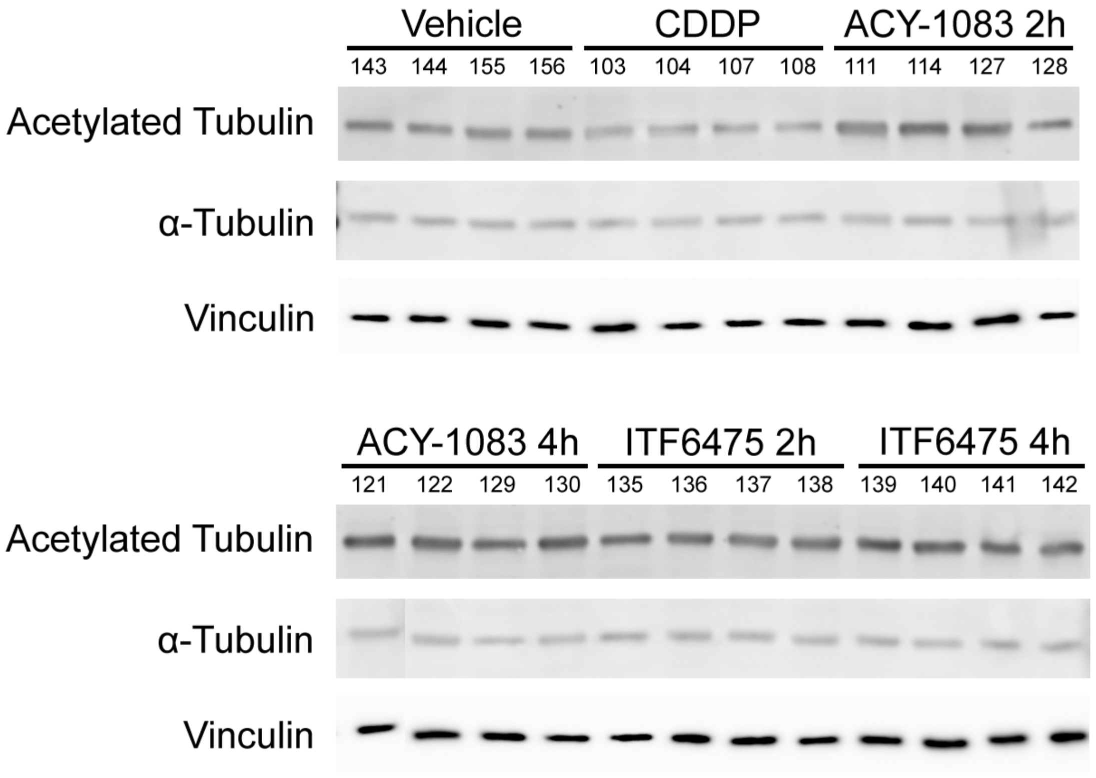

Figure 2S. Original western blot referred to Figure 12.
